# Leadership Hijacking in Docker Swarm and Its Consequences

**DOI:** 10.3390/e23070914

**Published:** 2021-07-19

**Authors:** Adi Farshteindiker, Rami Puzis

**Affiliations:** 1 Software and Information Systems Engineering, Ben Gurion University of the Negev, Beer Sheva 8410501, Israel; 2 Telekom Innovation Labs, Ben Gurion University of the Negev, Beer Sheva 8410501, Israel

**Keywords:** Docker Swarm, leader election, privilege escalation, defense evasion, cloud

## Abstract

With the advent of microservice-based software architectures, an increasing number of modern cloud environments and enterprises use operating system level virtualization, which is often referred to as container infrastructures. Docker Swarm is one of the most popular container orchestration infrastructures, providing high availability and fault tolerance. Occasionally, discovered container escape vulnerabilities allow adversaries to execute code on the host operating system and operate within the cloud infrastructure. We show that Docker Swarm is currently not secured against misbehaving manager nodes. This allows a high impact, high probability privilege escalation attack, which we refer to as leadership hijacking, the possibility of which is neglected by the current cloud security literature. Cloud lateral movement and defense evasion payloads allow an adversary to leverage the Docker Swarm functionality to control each and every host in the underlying cluster. We demonstrate an end-to-end attack, in which an adversary with access to an application running on the cluster achieves full control of the cluster. To reduce the probability of a successful high impact attack, container orchestration infrastructures must reduce the trust level of participating nodes and, in particular, incorporate adversary immune leader election algorithms.

## 1. Introduction

Securing distributed collaborative multi-agent agent systems is an extremely complex task. Since attackers are not obliged to follow the protocols defined by the system developers, they may create diverse adverse effects with simple manipulations applied to non-adversary-resilient protocols. Unfortunately, it is extremely difficult to secure a multi-agent system if it was not designed with security in mind. A good example of a design decision that may affect the overall security of a system is the choice of the leader-election algorithm [1]. In this article, we explore the consequences of the insecure leader election algorithm used in Docker Swarm.

**Figure 1:**
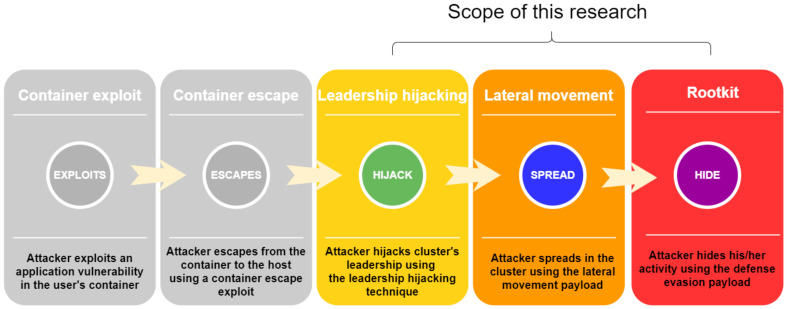
High-level description of the end-to-end scenario.

As Docker gained popularity among cloud service providers, attackers began to develop various techniques to attack Docker-based applications. Although a great deal of attention was paid to securing Docker hosts from application level exploits and container escape few solutions exist for securing against privilege escalation among different hosts in a Docker cluster. In this work, we show how an attacker with access to a manager host inside a Docker cluster can escalate their privileges in the cluster. The research scope is presented in Figure 1.

For example, Raft, a consensus algorithm used to manage a replicated log [2], is used in Docker Swarm to synchronize the cluster’s state between all managers of the cluster. See Section 2.2 for details. The logs are replicated using a strong leader, which is elected in the leader election phase in the algorithm. In case of a leader failure (a crash, network issues, etc.), the rest of the managers choose a new leader using the Raft algorithm. Despite its many advantages, Raft is a non-Byzantine algorithm that can allow a malicious insider to become a leader.

In this paper, we highlight a new privilege escalation technique called *leadership hijacking* (see Section 4.2). An attacker with access to a manager node in Docker Swarm can use this technique, which abuses the aforementioned fact that Raft is a non-Byzantine algorithm, to escalate their cluster privileges and become the cluster leader. By doing so, the attacker can control all messages and decisions within the cluster.

In addition, we demonstrate two possible malicious payloads expected to be executed by a typical attacker: a *lateral movement* payload and a *defense evasion* payload. The former utilizes cluster leader privileges and allows the attacker to execute code on every host in the cluster.

The latter is used by an attacker in order to hide their malicious activity from infrastructure management tools.

The rest of this paper is structured as follows: Section 2 reviews the technical background. In Section 4.2, we introduce the novel privilege escalation technique, called leadership hijacking. Next, in Section 4.3 we investigate malicious payloads that can be executed after the privilege escalation. In Section 5, we demonstrate an end-to-end attack scenario that illustrates the potential security risk and the impact of the investigated attack. Finally, in Section 6, we discuss possible mitigation and propose countermeasures. Our final remarks can be found in Section 7.

## 2. Background

### 2.1. Docker Swarm

An increasing number of organizations are moving their digital systems to the cloud. The benefits of cloud servers are easy deployment, high availability, continuous maintenance, system security, and more. From online websites to internal servers and databases, cloud servers store a lot of sensitive information, making them an attractive target for attackers. As the cost of hardware has decreased, software has become the main performance bottleneck. In order to fully utilize the available hardware, cloud service providers use virtualization technology to run different applications on the same hardware.

Until recently, the most advanced solution was virtual machine (VM) technology.

VM technology allows one physical server to run many different virtual servers, all of them running different operating systems.

From a security point of view, a VM is a good solution, since breaking out of a VM is a relatively complex task [3].

On the other hand, VMs suffer from significant performance overhead [4]. The main reason for the reduced performance is the overhead added by the hypervisor to each hardware operation emulated to the VM.

Today, many cloud service providers use operating system level virtualization, which employs isolated user space instances called containers. In contrast to a VM, which includes its own operating system, containers run under the host’s operating system and communicate with it directly. During the runtime, a container communicates through a regular system call interface with the host OS, without any intermediate software.

**Figure 2:**
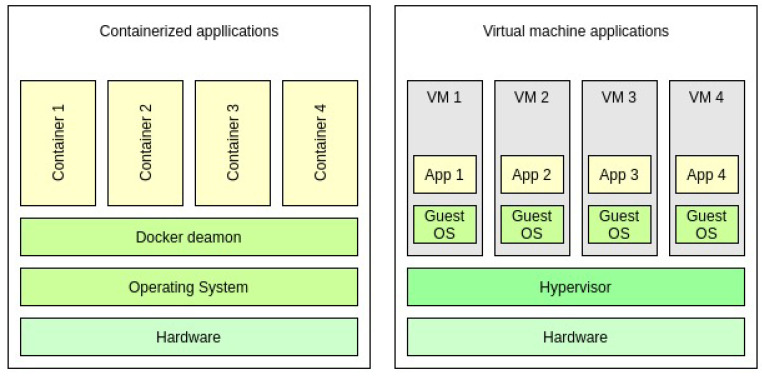
Container vs. VM architecture [5].

The architectural difference is illustrated in Figure 2.

At the time of this writing, Docker is one of the leading OS virtualization solutions (https://resources.flexera.com/web/media/documents/rightscale-2019-state-of-the-cloud-report-from-flexera.pdf (accessed on 8 July 2021)). Docker is implemented in the Go programming language and enables the creation, deployment, and management of containers on a host computer. A Docker container is a lightweight software unit that bundles its own tools and libraries. Typically, one container includes one instance of an application or service, e.g., a Web server, database, or scientific software package.

Docker is a rich ecosystem. One of the main components of this ecosystem is the Docker daemon. The Docker daemon is software that runs on the host and is responsible for the creation of images and containers. The Docker daemon can run containers and create their runtime environment; it can also create a container’s networking interfaces, mount points, can trigger actions, and execute commands inside a running container. The Docker daemon implements Docker’s main logic and many of its features.

When deploying an application in a production environment, it is important to ensure that when a container fails, a new container will start and replace the faulty container. In addition, it is highly recommended to run several instances of a container for high availability and load balancing. To address these issues, Docker introduced a feature called swarm.

Docker Swarm abstracts many Docker hosts to one virtual Docker host. Each host that participates in the swarm cluster is called a *node*. Each node can have two roles: *manager* or *worker*. A manager’s job is to keep a replicated state of the cluster. One manager node is also a *leader*. The cluster’s leader is responsible for scheduling new containers and services for the cluster. A worker’s job is to get container tasks from the leader and to actually run the container. The weakest point in the design of Docker Swarm exploited in this research is the Raft leader election algorithm.

### 2.2. Leader Election

Raft [2] is a consensus algorithm used to manage a replicated log. Raft was designed with the aim of producing an efficient and understandable algorithm which, unlike Paxos [6,7,8], would be easy to learn and use in practical systems. Raft was chosen in Docker Swarm due to its important features:

- Strong leader—Raft uses a stronger form of leadership than other consensus algorithms. For example, log entries only flow from the leader to other servers.
- Leader election—Raft uses randomized timers to elect leaders. This adds only a small number of mechanisms to the already existing heartbeat mechanism and facilitates simpler conflict resolution.
- Membership changes—Raft’s mechanism for changing the set of servers in the cluster uses a new joint consensus approach, which allows the cluster to continue operating normally during configuration changes.

Raft assumes that all nodes are honest and is not tolerant to malicious (Byzantine) nodes participating in the leader election process.

Byzantine fault tolerant (BFT) leader election algorithms have existed for a long time. These algorithms provide the ability to overcome failures in networks where some nodes are Byzantine. For example, Castro et al. [9,10] described a state machine replication algorithm able to tolerate Byzantine faults. The algorithm guarantees safety, i.e., each replicated log is agreed on by all non-faulty nodes.

Bessani et al. [11] introduced an open-source Java library implementing robust BFT state machine replication. Key features of their implementation include reliability, modularity, and a flexible application programming interface (API). Moreover, their implementation achieved good performance and can tolerate real world faults.

Castro et al. [9] implemented a BFT library, that can be used to build highly available systems that tolerate Byzantine faults. Castro et al. used the library to implement a Byzantine-fault-tolerant NFS file system. They showed that the replicated library can be even more efficient than the non-replicated version of NFS.

## 3. Related Work

When attacking a cloud based application, an adversary may exploit classical application vulnerabilities, such as SQL injection, buffer overflow, command injection, etc. Using such vulnerabilities, an attacker can control the victim’s container and data inside it. Container escape exploits are another technique class; in this case, after successful container exploitation, the attacker exploits a vulnerability allowing the attacker to escape from the container to the underlying host. Access to the underlying host grants an attacker access to data and other containers that run on the compromised host.

There are many products and protocols that try to mitigate the above-mentioned techniques. First, Docker offers built in protections (https://docs.docker.com/engine/security/ (accessed on 8 July 2021)), such as protecting the Docker daemon socket and using data encryption between the Docker daemon and public registries. These protections harden Docker hosts with a “security in depth” approach. In addition, software, such as SE-Linux and App-Armor, can help harden container isolation and minimize the attack surface between containers and the host. Furthermore, Docker offers an image scanning service (https://docs.docker.com/engine/scan/ (accessed on 8 July 2021)), which can detect vulnerabilities in Docker images.

In the rest of this section, we overview the previous work on cloud security related to Docker. Table 1 summarizes the main differences from related works.

Singh et al. [12] demonstrated primary techniques used by attackers to attack cloud services. There are many potential attack vectors that attackers can use, including: DoS and DDoS attacks [13,14], malware injection, and side-channel attacks [15,16,17,18]. In their study, Jensen et al. [19] demonstrated an attack on the software of the cloud itself and outlined the threat of flooding attacks on cloud systems. The authors suggested improving the cloud’s security by first improving the security of frameworks used in the cloud.

In [20], Liu et al. provided an overview of the latest technologies in cloud computing and discussed how Docker is integrated into it. According to Liu et al., the major difference between classic VM and containers is that a VM contains not only the application and its dependencies but also the entire guest operating system. The authors listed rapid application deployment, portability across machines, lightweight footprint, and minimal overhead as the main advantages of Docker over traditional VM-based virtualization software. Moreover, in [21], Marathe et al. overviewed the process of the setup of a computer cluster based on Docker Swarm and Kubernetes and evaluated each one of these platforms.

Xavier et al. [22] performed numerous experiments in order to evaluate the performance of container-based cloud environments compared to VM-based cloud environments as well as the trade-off between performance and isolation. They found that the cloud environment would benefit from container-based solutions, due to the fact that container-based solutions achieve near-native performance.

**Table 1:** Comparison with related works.

	Application Exploit	Container Escape Exploit	Cloud/Docker’s Framework Exploit	Orchestrator’s Exploit	Full Chain Attack POC	Attack Surface Overview
Our paper	✓	✓		✓	✓	
Wu et al., 2020 [23]	✓	✓				✓
Linetsky et al., 2020 [24]		✓	✓			
Seather 2018 [25]		✓				
Amara et al., 2017 [26]	✓	✓				
Kabbe 2017 [27]	✓					
Combe et al., 2016 [28]	✓		✓			
Singh & Shrivastava 2012 [12]	✓					✓
Jensen et al., 2008 [13]			✓			

Other research [28] suggested a new attack surface in the Docker environment: namely indirect adversaries. Unlike a direct adversary, who exploits vulnerabilities in the cluster directly, an indirect adversary exploits third party appliances (e.g., Docker Hub) in order to attack Docker’s environment.

An overview of attack types and mitigations in cloud environments is shown in [26]. Among others, Amara et al. mentioned SQL injection as “application level attack”, which is used to obtain an initial foothold in the cluster. Moreover, they mentioned hypervisor attacks as “VM level attacks”, which are used for privilege escalation and breaking VM isolation. In addition, they offered mitigations to each one of the attacks that they describe.

Moreover, Wu et al. [23] evaluated the security of container based cloud services. They defined metrics upon which they evaluated a number of services. Among others, they specified “privilege escalation” metric and “container escape” metric. They found that, although there are some services that failed in the “privilege escalation” metric, the “container escape” metric was very high, which limits the impact of the attacker.

In his master’s work, Kabbe [27] compared the security model of containers to hypervisor-based systems and virtual machines. He compared the outcome of known attacks (DirtyCow, (https://nvd.nist.gov/vuln/detail/CVE-2016-5195 (accessed on 8 July 2021)) Heartbleed, (https://nvd.nist.gov/vuln/detail/CVE-2014-0160 (accessed on 8 July 2021)) and Shellshock (https://nvd.nist.gov/vuln/detail/CVE-2014-6271 (accessed on 8 July 2021))) in a containerized environment, with the outcome of the same attacks performed in hypervisor/virtual machine environments. He found that containers offered at least the same amount of security as hypervisor/virtual machine environments.

In his master thesis [25], Seather reviewed the underlying security of the Docker Swarm infrastructure. Namely, Seather tested many adversarial scenarios, including: flooding the orchestrator with invalid/corrupted requests, sniffing the network from within the cluster, impersonating a cluster member, performing man-in-the-middle attacks between containers within Docker’s internal network, and more. The conclusions of his thesis were that Docker’s infrastructure is secure, Docker Swarm’s design is good (from a security point of view), the technology stack used by Docker is immune to known attacks, and the development community responds quickly to security incidents.

Attacking the cloud’s infrastructure is also shown in [24]. In their work, Linetskyi et al. showed and utilized a Kubernetes privilege escalation exploit, in which an attacker can obtain a root privileges inside a container. If the container is misconfigured, this can result in root privileges to the underlying host. The bug resides in Kubernetes’s management tool, which stresses the fact that extra care should be made to secure the code of the infrastructure (in that case, Kubernetes).

## 4. Taking over the Docker Swarm

In this section we present the new techniques that can be used to take over a Docker Swarm cluster. We present a full exploit chain starting with existing container escape exploit. When combined with our leadership hijacking technique it ultimately gives the attacker cluster leader privileges. Later, we show how our malicious payloads can be used to completely compromise cloud environment while evading detection.

### 4.1. High-Level Overview

A high-level overview of the end-to-end attack scenario can be seen in Figure 1. The attack consists of five major steps:

1. Exploitation of an application vulnerability inside a container, in which an attacker gains a foothold within the user’s container
2. Container escape exploitation, in which an attacker obtains access to the container’s underlying host
3. Leadership hijacking, in which an attacker executes the privilege escalation technique presented in Section 4.2 and obtains cluster leader privileges
4. Lateral movement, in which an attacker executes the lateral movement payload described in Section 4.3.1 and gains privileged access to all hosts in the cluster
5. Defense evasion, in which an attacker uses the defense evasion payload described in Section 4.3.2 in order to hide their lateral movement payload from management tools

In order to demonstrate the feasibility and impact of the leadership hijacking technique and the malicious payloads, we developed an end-to-end attack scenario that shows how an external attacker can chain exploits seen in the wild with our technique and payloads, in order to obtain full control of a cluster. A detailed description of this scenario is provided in Section 5. Steps 1 and 2 are implemented in order to demonstrate the feasibility of our work, but they are not elaborated upon, since they are out of the scope of our research.

### 4.2. Leadership Hijacking

In this section, we introduce an adversarial technique named leadership hijacking. A precondition to employing this technique is code execution access to a manager node.

In Section 5, we show how this precondition can be achieved in a production environment. From now on, we will refer to the manager host compromised by the attacker as the attacker’s manager. The main idea of our technique is to repeatedly trigger a leader election phase until the attacker’s manager becomes the cluster leader.

The technique’s pseudocode is shown in Algorithm 1.
**Algorithm 1** Attack pseudo code.  1:@Pre-condition: attacker escaped his container  2:**procedure**Get-Leadership  3: **if** Attacker’s manager is cluster leader **then**  4:  Exit  5: **while** Attacker’s manager is not leader **do**  6:  leader_id← find out current leader ID  7:  demote node with id leader_id  8:  wait until a new leader is elected  9:  promote node with id leader_id to be manager10:@Post-condition: attacker’s manager is the leader

As shown in Algorithm 1, the first step of the technique is to identify the current cluster leader. If the current leader is the attacker’s manger, the technique’s code will exit. Otherwise, the technique starts a loop.

In each loop iteration, the technique demotes (i.e., removes from the leader role) the current cluster leader using the Docker’s demotion API [29]. This will cause the cluster to initiate a leader election algorithm and elect a new leader. The first manager that reaches timeout proposes itself as the cluster leader. Afterwards, each manager votes in favor of one manager, and the manager that receives the majority of the votes becomes the new cluster leader.

In the final step of the iteration, the current cluster leader is identified again. If the attacker’s manager is the leader, the technique exits. Otherwise, it will continue the loop until the attacker’s manager becomes the cluster leader. To avoid being detected through repeated reduction in the number of available managers, the attacker promotes the demoted node back to the manager role [30] by the end of each leader election.

**Figure 3:**
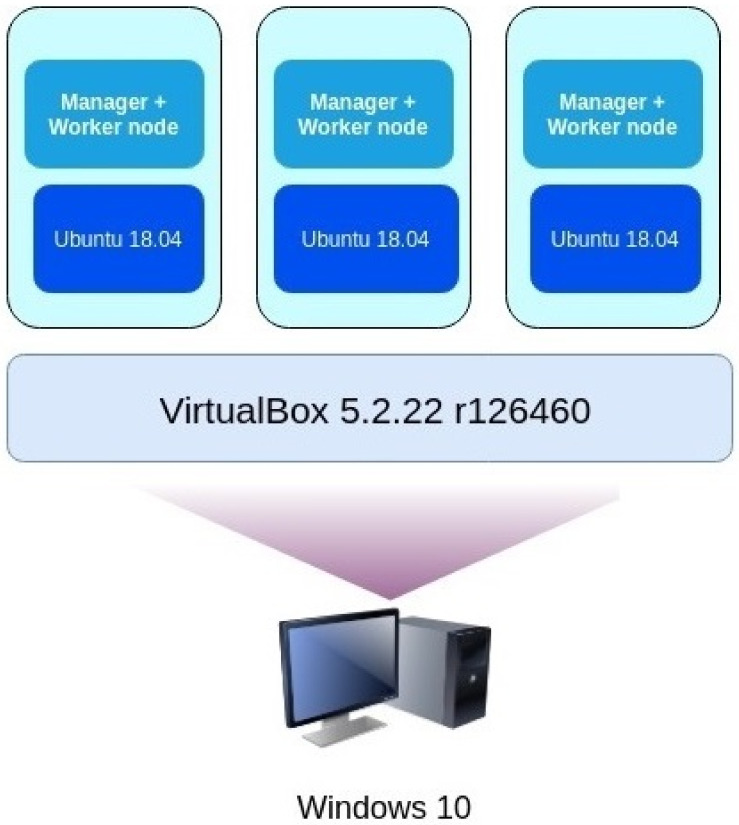
Overview of the lab architecture.

In order to prove that the technique works in practice, we implemented the pseudocode shown in Algorithm 1. We set up a lab to test the implementation, and its architecture is illustrated in Figure 3.

Running our technique’s implementation in the lab was successful: the attacker was able to escalate privileges in order to become the new cluster leader.

#### 4.2.1. Analysis

##### Convergence

In each iteration, the technique code demotes the leader. According to the Docker Swarm documentation, a manager that does not receive the heartbeat from the leader during the predefined time window assumes that the leader is unavailable and proposes itself to be the new cluster leader. Since the leader has been demoted, none of the managers receive the heartbeat from the leader, and hence a new leader election phase will start when the first manager reaches its timeout.

Docker Swarm closely follows the specification and implementation of Raft where the election timeout (the time a node waits before starting a new election) is randomly drawn from a predefined range. In addition to the election timeout, the probability of every manager becoming a leader depends on the communication delays and may not be the same for all managers [31]. Yet, it is safe to assume that in a properly configured swarm, every manager has a roughly equal probability to be elected.

In the absence of an attacker, each leader election is independent of the previous iterations of leader election. This stems from the fact that Raft nodes do not maintain any state concerning the leader election process except being a follower, a candidate, or a leader (Temporarily, there may be more than one node in a leader state due to collisions, which are solved by Raft). The attack introduces a slight dependency between iterations due to the absence of the previous demoted leader in the set of candidates.

The absence of a candidate cannot reduce the probability of the attacker’s manager being elected. Thus the probability of the attacker’s manager to be elected during each attack iteration is bounded from below by the probability of the respective manager to be elected without the attack. The positive probability of the attack success in each iteration and the ability of the attacker to continue demoting the leaders guarantee the eventual success of the attack.

The positive probability of the attack success in each iteration and the ability of the attacker to continue demoting the leaders guarantee the eventual success of the attack. In a properly configured system where each manager has the same probability to be elected, the number of managers is the mean number of leader elections until the attack succeeds.

##### Advantages

The first advantage of the technique is its simple implementation. In order to prove its feasibility, we decided to implement the technique in the most simple way possible. After reviewing the Docker Swarm API, we realized that our technique could be implemented with repeated calls to demote and promote API [29,30]. This simple implementation makes our technique stable and reliable.

The second advantage of our technique is its stealthiness. A typical attacker would like to stay undetected as long as possible while in an engagement. Our technique can be implemented in many ways; however, some are rather loud, which will increase the chance to get caught by the system administrators. For example, an attacker can demote all other managers of the cluster and become the only manger and, hence, the cluster leader. The obvious issue of this implementation is that the system administrators will quickly notice that the cluster state has changed. On the other hand, our implementation’s changes to the cluster state are minimal, which makes it harder to detect the technique.

##### Limitations

The main limitation of our technique is that it is probabilistic. Although we showed that our technique completes successfully with probability P→1, the number of iterations in each execution may differ. An unknown number of iterations is particularly problematic in a real-world scenario.

### 4.3. Malicious Payloads

In order to illustrate the impact of the leadership hijacking technique, we developed malicious payloads that use cluster leader privileges and used them to perform some malicious operations.

Typically, an attacker who has access to one host inside a cluster would like to spread and obtain a wider foothold in the cluster. Ideally, the attacker would like to have access to all hosts in the cluster, with high privileges in each host. Moreover, once the attacker controls a cluster they would like to remain undetectable by the users/system administrators for as long as possible.

To achieve the above goals, the attacker has to find a way to spread inside the cluster and hide their malicious activity from users and monitoring tools. In this work, we introduce and develop two types of malicious payloads: a lateral movement payload and a defense evasion payload. These payloads utilize leader privileges and allow an attacker to execute high privileged code on every node in the cluster and hide from monitoring tools.

#### 4.3.1. Lateral Movement

Typically, an attacker would like to establish a wide foothold in a cluster, preferably with high privileges. In this work, we create a payload that enables lateral movement in the cloud. Using this payload, we demonstrate how an attacker with leader privileges in a Docker Swarm cluster can execute high privileged code on each host in the cluster.

Due to the fact that, after successful execution of leadership hijacking, the attacker gains leader privileges, the attacker can control all messages that come out of the leader node. By hooking the leader’s function responsible for sending messages between the leader and other nodes, the attacker can change these messages and alter their content.

In order to execute code on other nodes in the cluster, the attacker who is in control of a leader host can send the victim node a task to run. The attacker instructs the worker to run a container task with an image controlled by the attacker. As we show in Section 5, the victim node will execute the container. The container’s image will be a malicious image.

However, the malicious container runs in an isolated environment in the host. As discussed in Section 3, containers run in a separate namespace from the host. Thus, for example, a process inside a container cannot sniff the host’s network.

There are many ways to overcome this limitation. In addition to controlling what image the container will run on each host, the attacker also controls the creation flags of the container. Thus, for example, the attacker can mount the main file system of the host to the container. Then, from inside the container, the attacker can alter the host’s executable files with a malicious code. In order to obtain highly privileged code execution, the attacker has to alter a file that is executed by a highly privileged user on the host. When the user executes the file, the attacker’s malicious code will get executed as well, resulting in high privileged code execution on the host.

#### 4.3.2. Defense Evasion

With the above lateral movement payload, the attacker can spread and move laterally by deploying service with malicious image to every host in the cluster. In this subsection, we show how an attacker can stay undetected in the cluster and hide malicious activity from the cloud’s management tools. We introduce the cloud defense evasion payload, which offers rootkit-like functionality in the cloud.

In this subsection, we assume that the attacker is the cluster leader and has a malicious service in the cluster, which they wish to hide from system administrators, e.g., a malicious cryptocurrency mining service.

The default Docker Swarm command line offers a rich variety of commands for cluster administration. In particular, Swarm offers the docker service (https://docs.docker.com/engine/reference/commandline/service/ (accessed on 8 July 2021)) command for viewing and updating services that run on the cluster. In order to view services that run on the cluster, the system administrator can issue the docker service ls (https://docs.docker.com/engine/reference/commandline/service_ls/ (accessed on 8 July 2021)) command and view its output. The output includes the service’s name, image, number of replicas, exposed ports, etc.

In order to obtain this information, the Docker daemon of the host that issued the command queries the leader of the cluster and retrieves the information from the leader.

However, the attacker is in control of the leader host. Hence, the attacker can hook the function that returns this information on the leader’s Docker daemon and spoof the answers. In this way, the attacker can change malicious service’s name, image, ports, or even the service itself (i.e., the attacker can trick the user into thinking that there is no such service at all, by removing any information related to the malicious service).

In a similar manner, the system administrator can view what containers are running for each service. Using docker service ps (https://docs.docker.com/engine/reference/commandline/service_ps/ (accessed on 8 July 2021)) command, the system administrator can obtain information about a container’s image, name, state, etc. In a similar way to the docker service ls command, the issuing host queries the leader host and retrieves that information. The attacker has access to the leader host, and thus they can alter that information as well. By doing so, the attacker can trick the system administrator and show them that a container is running a different image than the real image, for example.

In this way, the attacker can hide malicious activity from Docker’s default tools, which query the cluster leader to obtain information about objects (running services, containers, etc.) in the cluster.

## 5. End-to-End Attack Showcase

To prove that our leadership hijacking technique and malicious payloads are feasible, we implemented a combined scenario that demonstrates the impact of our technique and of the payloads. We show the importance of our technique and payloads, as well as that the initial assumption regarding the attack is reasonable. We provide proof-of-concept demonstration of an external attacker leveraging an exploit, which has been seen in the wild together with our leadership hijacking technique and malicious payloads, in order to ultimately control the entire cluster.

### 5.1. Lab Setup

To demonstrate the attack we set up a test-bed that, on the one hand, mimicked a cloud environment with a Docker Swarm and multiple client’s services; and, on the other hand, included a typical attackers’ tool set.

Cloud nodes were simulated using virtual machines that ran the Ubuntu guest OS. We set up a Docker Swarm cluster in which all hosts were both manager and worker hosts. In addition, an external laptop was used as the attacking machine. The laptop ran the Kali Linux operating system version 2019.3.

One important tool that we used was the Metasploit framework [32], an open-source framework supporting various penetration testing tasks.

**Figure 4:**
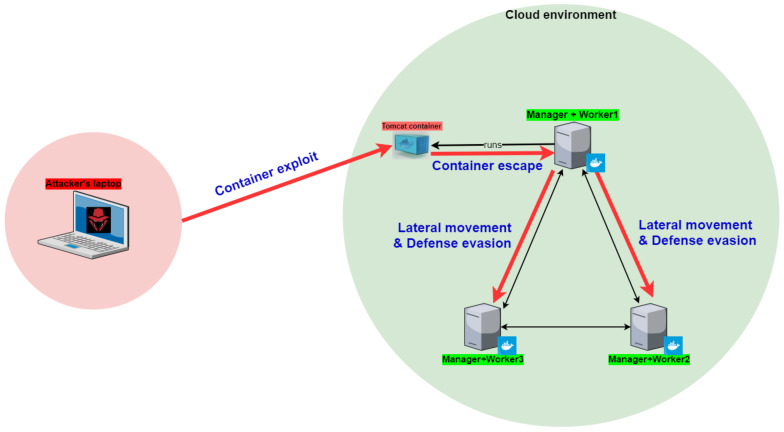
Diagram of the attack steps.

The lab’s architecture is shown in Figure 4.

### 5.2. Scenario Overview

In our end-to-end attack scenario, the attacker started on an external laptop with network access to a Docker container that ran inside a Docker cluster. Ultimately, the attacker obtained high privileged code execution on each host in the cluster. The scenario contained five major steps:

1. Container exploitation
2. Container escape exploitation
3. Leadership hijacking
4. Lateral movement
5. Defense evasion

In each step, the attacker expands their foothold in the cluster. An illustration of the entire scenario and its steps can be seen in Figure 4.

The next subsections explain these steps in greater detail.

### 5.3. Container Exploitation

First, the attacker needs to have an initial foothold in the cluster. They have network access to an application that runs on a container in the cluster. In order to obtain an initial foothold, the attacker exploits a vulnerability in the application.

In this case, the application running inside the container is the Apache Tomcat Web server, version 8.5.19. The attacker finds a one-day exploit for that Web server in the Metasploit framework; after successful exploit completion, the attacker has shell access to the application’s container.

### 5.4. Container Escape

After the attacker has successfully exploited the application, the attacker has a shell in the restricted Docker environment. In order to execute our privilege escalation technique, the attacker needs to escape from the restricted environment and retrieve a shell on the underlying host of the container.

The attacker then exploits a vulnerability in the host’s RunC component (https://www.cvedetails.com/cve/CVE-2019-5736/ (accessed on 8 July 2021)). RunC is a container runtime that was originally developed as part of Docker, which is responsible for running and managing new container environments.

A vulnerability resides in RunC version < 1.0-rc6 (which is used by Docker < 18.09.2), allowing the attacker to overwrite the host’s RunC binary and, thus, achieve code execution with root privileges on the host.

### 5.5. Cloud Privilege Escalation

Once the attacker has achieved code execution on Docker’s manager host, they can execute the leadership hijacking technique and escalate their privileges in order to become the cluster leader (see Section 4.2 for a description of the leadership hijacking technique).

After the leadership hijacking technique’s successful execution, the attacker obtains leader privileges in the cluster and, thus, will be able to control all messages that flow between the leader and other hosts in the cluster.

**Figure 5:**
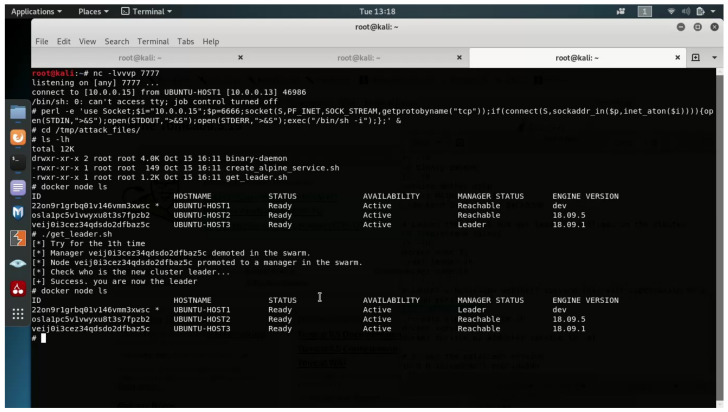
Successful attack attempt.

The result of the technique’s successful execution can be seen in Figure 5. In this figure, we can see that, before the attack, UBUNTU-HOST3 was the cluster leader, and after the technique was successfully executed, UBUNTU-HOST1 (which is the attacker’s manager) obtained the leadership role in the cluster.

### 5.6. Lateral Movement and Defense Evasion

Armed with leader privileges, the attacker can now control all messages that flow between the leader and other hosts in the cluster. As described in Section 4.3.1 and Section 4.3.2, the attacker can execute a malicious container on each host in the cluster and hide these actions from various management tools.

To effectively demonstrate the attack and its potential impact, in our scenario, the attacker will run a WebShell service, which will run a WebShell container on every host in the cluster.

The malicious WebShell container provides a root privileged command execution environment on the underlying host. The host’s file system is mounted in the container’s /tmp directory. This allows the attacker to view, modify, and delete the host’s files. Effectively, the attacker runs a root WebShell on all hosts in the cluster.

**Figure 6:**
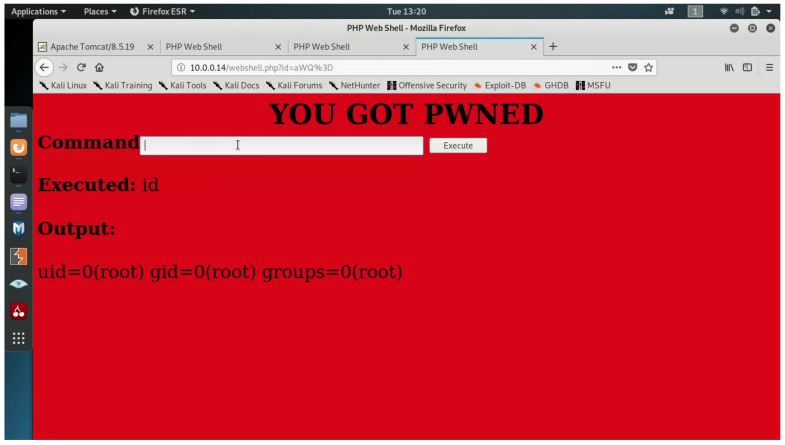
The output of the malicious WebShell.

The output of the WebShell can be seen in Figure 6. In addition, the figure shows that the WebShell is executed with high privileges (root).

The attacker uses the defense evasion functionality described in Section 4.3.2, hooking the leader’s Docker daemon function, which is responsible for listing the services and containers of services. By doing so, any service listing request that is made to the cluster leader will be monitored by the attacker. In cases in which the attacker’s malicious service is running, the attacker will spoof the answer of the listing and hide their malicious service image with a benign Alpine image.

**Figure 7:**
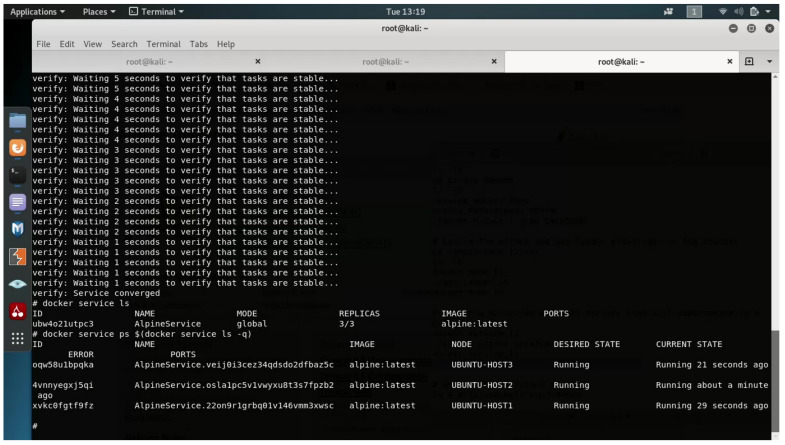
Docker’s default tools used for viewing information about malicious services.

As seen in Figure 7, docker service ls command reveals a single running service, with image "alpine:latest". In addition, it seems that there are no listening ports; however, in actuality, a container on each host is listening on port 80.

Furthermore, the attacker also hooks the function responsible for listing container of each service; thus, the output of docker service ps $(docker service ls -q) does not reveal the real image that each container is actually running. According to Docker’s default tools, it looks like the service running is a benign alpine service but accessing each host in port 80 reveals the true “face” of the service.

## 6. Discussion

The main advantage of our technique is that, unlike many techniques seen in the wild, our technique does not exploit any software bugs. A software bug is usually a mistake in a program’s code, which can lead to an undefined behavior of the program. In most cases, software bugs are easily fixed. However, our technique does not exploit any programming errors but rather exploits a design flaw. Unlike programming bugs, logical bugs are much harder to fix, since, in many scenarios, a large amount of code must be changed, which can be costly and time-consuming for software developers.

As shown in Section 4.2, our technique exploits the fact that the Raft algorithm is used to replicate logs in the Docker Swarm environment but is a non-adversarial algorithm. Raft is a key component of Docker Swarm’s management infrastructure, and it is integrated into the core logic of Docker Swarm. Replacing the Raft algorithm in Docker Swarm is a mandatory step to mitigate our proposed technique, since exploits used to escape from container to host (as shown in Section 5) are very common and relatively easy to find. Since its a design bug, replacing Raft requires a significant amount of work.

First, Docker’s developers should choose and implement a byzantine fault tolerant algorithm [9,11] in Go, or find such an implementation as a Go package. The implementation should be high quality, since it will be deployed to every manager in the cluster. Next, the developers should modify Docker Swarm’s source code. In Docker Swarm, Raft’s implementation is encapsulated with a wrapper object. The developers of Docker Swarm should change the entire wrapper object to encapsulate the new package instead of Raft.

Then, series of tests should be ran to ensure that the new package meets Docker’s efficiency requirements: both local and network. The new package should not consume a significant amount of the host’s resources, and should be be efficient in terms of network activity between hosts in the Docker Swarm. Moreover, the tests should ensure that the new package works as expected on every operating system supported by Docker Swarm. Since managers are the most valuable servers in the cluster, any bug in a manager can be fatal. The tests should ensure, as much as possible, that the new package is bug free and that it has no unwanted side effects. In any case, replacing the Raft implementation holds a major risk and may cause a service degradation.

There are some best practices that may block our attack; the most common is to separate the manager nodes from worker nodes. In such a case, even if the attacker compromised a worker node, he will not be able to escalate his privileges in the way we suggested in this article, since the attacker’s node is not part of the managers group. However, although considered a best practice, this is not the default behavior of Docker Swarm. We believe that Docker’s developers chose to make the manager node a worker too by default in order to not waste expensive computing power. If a node is just a manager, it will not receive the client container to execute, and hence the cluster’s computing capacity decreases. Regardless, in this article, we chose to research and exploit systems in their default state and not delve into best practices.

We offer two strategies in order to effectively mitigate our technique. In the short term, the technique can be mitigated by detecting and blocking container escape exploits. As discussed in Section 4.2, the leadership hijacking technique should be executed from a manager host. We showed in Section 5 that an attacker can gain such access using a container escape exploit. In the case that the container escape exploit fails, an attacker cannot launch the technique and, therefore, cannot escalate his privileges in the cluster. In order to reduce the amount of container escape exploits, Docker can start a bug bounty program. We believe that this will help Docker patch container escape vulnerabilities before they can be exploited by real attackers in the wild.

In the long term, we offer to replace the Raft algorithm with a byzantine fault tolerant algorithm [33,34]. As discussed earlier, Raft is a non adversarial algorithm; hence, an attacker who is in control of a Raft’s participant can forge and spoof messages. In that way, the attacker can trick other participants to vote for him in the leader election phase and become the cluster’s leader. In the case that a BFT algorithm is used, other participants would not vote for the attacker since the algorithm can tolerate byzantine participants. In that way, the attacker would not be able to escalate his privileges to cluster leader. Furthermore, in order to support future changes, the developers of Docker should divide Docker’s infrastructure from the leader election algorithm. The architecture of Docker Swarm should be “plug and play”, such that the leader election algorithm is chosen as a configuration option instead of a source code modification.

## 7. Conclusions

In this work, we suggested a new attack vector on the Docker Swarm orchestrator. Our technique demonstrated a new concept in offensive security in which a cluster is treated as a single unit of processing and an attacker is able to escalate their privileges in that unit and, thereafter, perform malicious activity on every component of that unit separately (i.e., every host in the cluster).

We presented a novel technique that, when combined with our proposed payloads, allows an attacker to gain full control over the Docker Swarm cluster. Since our technique and payloads do not exploit a software bug but rather exploit a design weakness, developers should take them into account during the design of their multi-agent systems. Future research should, on the one hand, explore additional ways in which attackers can obtain leader privileges in other cloud environments, e.g., Kubernetes, and, on the other hand, develop methods to detect misbehaving managers, for example, using anomaly detection techniques.

## Data Availability

Not applicable.
